# Identification of predictive genetic signatures of Cytarabine responsiveness using a 3D acute myeloid leukaemia model

**DOI:** 10.1111/jcmm.14608

**Published:** 2019-08-26

**Authors:** Haiyan Xu, Eric S. Muise, Sarah Javaid, Lan Chen, Razvan Cristescu, My Sam Mansueto, Nicole Follmer, Jennifer Cho, Kimberley Kerr, Rachel Altura, Michelle Machacek, Benjamin Nicholson, George Addona, Ilona Kariv, Hongmin Chen

**Affiliations:** ^1^ Department of Pharmacology Merck & Co., Inc. Boston MA USA; ^2^ Department of Genetics and Pharmacogenomics Merck & Co., Inc. Boston MA USA; ^3^ Department of Strategic Planning & Research Informatics Merck & Co., Inc. Beijing China; ^4^ Department of Precision Oncology Biomarkers Merck & Co., Inc. Boston MA USA; ^5^ Department of Oncology Early Discovery Merck & Co., Inc. Boston MA USA; ^6^ Department of Oncology Early Development Merck & Co., Inc. Rahway NJ USA; ^7^ Department of Discovery Chemistry Merck & Co., Inc. Boston MA USA

**Keywords:** 3D culture, acute myeloid leukaemia, gene fusion, RNASeq, whole‐exome sequencing

## Abstract

This study reports the establishment of a bone marrow mononuclear cell (BMMC) 3D culture model and the application of this model to define sensitivity and resistance biomarkers of acute myeloid leukaemia (AML) patient bone marrow samples in response to Cytarabine (Ara‐C) treatment. By mimicking physiological bone marrow microenvironment, the growth conditions were optimized by using frozen BMMCs derived from healthy donors. Healthy BMMCs are capable of differentiating into major hematopoietic lineages and various types of stromal cells in this platform. Cryopreserved BMMC samples from 49 AML patients were characterized for ex vivo growth and sensitivity to Ara‐C. RNA sequencing was performed for 3D and 2D cultures to determine differential gene expression patterns. Specific genetic mutations and/or gene expression signatures associated with the ability of the ex vivo expansion and response to Ara‐C were elucidated by whole‐exome and RNA sequencing. Data analysis identified unique gene expression signatures and novel genetic mutations associated with sensitivity to Ara‐C treatment of proliferating AML specimens and can be used as predictive therapeutic biomarkers to determine the optimal treatment regimens. Furthermore, these data demonstrate the translational value of this ex vivo platform which should be widely applicable to evaluate other therapies in AML.

## 1 INTRODUCTION

Acute myeloid leukaemia is a disease of complex genetics. The prognosis is generally poor with the overall 5‐year survival rate less than 40% in adults. The survival rate is negatively associated with age and is less than 10% for patients older than 60 years.^1^, ^2^ If left untreated, AML progresses very rapidly and is fatal within months or even weeks. Acute myeloid leukaemia is a clonal myeloid lineage malignancy with more than 2000 gene mutations identified to date, including chromosomal abnormalities and a wide spectrum of gene mutations within a normal karyotype.^2^, ^3^, ^4^ Chromosomal translocations often cause gene fusions of various transcription factors with different partners and alter the expression of genes involved in the development of AML.^3^ Acute myeloid leukaemia classifications by recent WHO criteria are focused on significant cytogenetic and molecular genetic subgroups.^5^ Chemotherapy has remained as a main treatment for AML for the past several decades. Breakthrough new AML therapies of two targeted drugs, midostaurin and enasidenib, were approved by the US Food and Drug Administration (FDA) in 2017. Midostaurin targets AML harbouring fms like tyrosine kinase 3 (FLT3) mutations, whereas enasidenib treats AML with an isocitrate dehydrogenase (IDH) 2 mutations. Most recently Venetoclax, a Bcl‐2 inhibitor, has also been approved by the FDA to treat AML. Selection of appropriate patients for clinical trials has been a challenging task, and researchers have been constantly searching for genetic biomarkers and gene signatures that can be used to predict drug responsiveness by whole‐exome sequencing (WES) and RNAseq technologies.^4^, ^6^ Drug responsiveness was usually evaluated in 2D ex vivo culture during short‐term treatment of 2‐4 days using viability assay, which is considerably shorter than the duration of chemotherapy in the clinic that lasts ten days per cycle.^1^

The 2D culture has been recognized for a long time as an insufficient system to accurately predict drug responsiveness due to the lack of physiologically relevant microenvironment.^7^, ^8^ To overcome this limitation, 3D culture models have been developed in recent years for various cell types and tissues.^7^, ^8^ The 3D ex vivo models can recapitulate the complex physiology and maintain functional in vivo responses that are not observed in routine 2D cultures. In the case of 2D culture of leukaemic BMMCs, lack of bone marrow microenvironment prevents long‐term culture of leukaemic cells without supplementing maintenance media with growth factors or co‐culture with stromal cells. However, co‐culture with stromal cells further complicates drug testing because specific killing of AML needs to be carefully determined, and often stromal cell growth can become dominant unless irradiated stromal cells are used. In recent years, several 3D models have been reported for culturing bone marrow samples, including matrigel, hydrogel, synthetic polymers and human amniotic membrane with or without co‐culture of stromal cells.^9^, ^10^, ^11^, ^12^ The stiffness of matrix also plays an important role in AML cell growth and responsiveness to drug treatment. Shin and Mooney reported that matrix mechanics influenced both cell proliferation and sensitivity to chemotherapeutic agents of several AML cell lines ex vivo and in vivo using a hydrogel system.^13^ However, it remains to be investigated whether any of these 3D systems can be used to identify gene signatures and mutations predictive of responsiveness to drug treatment of BMMCs from AML patients.

In the current study, a 3D platform was established and evaluated to culture BMMCs from both normal donors and AML patients. Under optimized conditions, 3D bone marrow cultures properly maintained growth and showed differentiation of major hematopoietic cell lineages as well as stromal cells. Gene expression signatures of 2D and 3D cultures were evaluated by comparing to the cryopreserved BMMCs using RNAseq. This platform was then used to identify AML responders and non‐responders to Ara‐C, a first‐line chemotherapy drug for treating AML.^1^ Eight Ara‐C responders and five non‐responders were assessed for differential gene expression patterns in vehicle vs Ara‐C treatment. Furthermore, novel genetic mutations associated with Ara‐C responsiveness were revealed through WES. These results indicate that 3D ex vivo translational platforms can assist in identifying prospective biomarkers, accelerate discovery of novel treatments as well as to define AML treatment regimens in the clinical setting prior to initiation of therapy.

## 2 METHODS

### 2.1 Cells and reagents

Normal and AML patient BMMCs were purchased from Folio Conversant and ProteoGenex. Recombinant human fibronectin was purchased from Millipore. Rat tail collagen type 1 and matrigel were purchased from Corning Inc. CellTiter‐Glo^®^ 3D cell viability assay reagent was purchased from Promega. Human thrombopoietin, human interleukin‐3 (IL3), human stem cell factor, human granulocyte‐macrophage colony‐stimulating factor (GM‐CSF) and human macrophage colony‐stimulating factor (M‐CSF) were purchased from Life Technologies. Rosiglitazone, human erythropoietin (EPO), granulocyte colony‐stimulating factor (G‐CSF), interleukin‐7 (IL‐7) and receptor activator of nuclear factor kappa‐Β ligand (RANKL) were purchased from R&D systems. Human FLT3 ligand was purchased from TONBO Biosciences. Qubit™ dsDNA HS Assay Kit and MirVana miRNA isolation kit were purchased from Thermo Fisher. Organoid harvest media was purchased from Trevigen. RNeasy Plus Micro Kit, QIAshredder mini spin column and AllPrep DNA/RNA Mini were purchased from Qiagen. Agilent RNA 6000 Pico Kit was ordered from Agilent Technologies. FITC‐anti‐human MPO flow kit was purchased from BioLegend. FITC‐labelled anti‐human CD71, FITC‐labelled anti‐terminal deoxynucleotidyl transferase (TDT), and anti‐human CD110 were purchased from BD Biosciences. TRAP staining kit was purchased from B‐Bridge International, Inc.

### 2.2 Normal and AML BMMC culture

Bone marrow mononuclear cells from healthy donors or AML patients were thawed in FBS, filtered through 100 μM Nylon mesh, counted, centrifuged and re‐suspended at the density of 7 × 10^4^/µL in IMDM supplemented with the following components: 20% FBS, 620 μmol/L CaCl2, 1 μmol/L sodium succinate, 1 μmol/L hydrocortisone, 55 μmol/L β‐mercaptoethanol, antibiotic‐antimycotic, 100 ng/mL human SCF, 50 ng/mL human FLT3L, 20 ng/mL human IL3, 20 ng/mL human GM‐CSF, 100 ng/mL human M‐CSF, 20 ng/mL human G‐CSF, 20 ng/mL human IL‐7, 40 ng/mL human TPO, 1.5 U/mL human EPO and 100 ng/mL human RANKL. Ten volumes of 40% matrigel containing 333 µg/mL fibronectin and 266 µg/mL collagen I were added to cell suspension. After gentle mixing, cells were seeded in 384‐well plate at the density of 80 000 cells per well. For AML samples, a 15‐point 3‐fold dilution dose‐response curve starting with 10 μmol/L was performed by treating cells embedded in 3D gel on the second day with DMSO as the vehicle control and again 5 days later. Cell viability was assessed using CTG‐3D after 11 days of treatment. IC50 values were calculated using GraphPad Prism 7 by plotting the data through non‐linear regression after transforming X‐axis to log scale and normalizing CTG reading to DMSO‐treated control cells harvested on the same day. Among the 49 AML donors tested, only 23 IC50 curves were plotted for robust growing AML donors. Robust growth was defined as more than 50% confluency of the well when observed under microscope after 11 days of culture.

### 2.3 AML patient information

Acute myeloid leukaemia patient bone marrow mononuclear cells (BMMC) were purchased from Conversant Bio and ProteoGenex. A total of 49 AML BMMC samples were tested, and patient information is summarized in Table 1.

**Table 1:** AML patient information

Sample ID	Treatment	Gender	Race	Age	Blast %	Clinical subtype
Donor 1	Pre‐Treat	Male	Caucasian	70	99	N/A
Donor 2	Pre‐Treat	Male	Caucasian	84	90	N/A
Donor 3	Pre‐Treat	Male	Caucasian	79	80	M0‐1
Donor 4	Pre‐Treat	Male	Caucasian	57	80	N/A
Donor 5	Pre‐Treat	Male	Caucasian	36	100	N/A
Donor 6	Pre‐Treat	Female	Caucasian	45	80	N/A
Donor 7	Pre‐Treat	Female	Caucasian	79	100	N/A
Donor 8	Pre‐Treat	Female	Caucasian	73	92	M0
Donor 9	Pre‐Treat	Male	Caucasian	35	86	M1
Donor 10	Pre‐Treat	Male	Caucasian	77	92	N/A
Donor 11	Pre‐Treat	Female	Caucasian	48	82	M3
Donor 12	Pre‐Treat	Male	Caucasian	37	88.4	N/A
Donor 13	Pre‐Treat	Male	Caucasian	51	85	N/A
Donor 14	Pre‐Treat	Female	Caucasian	55	90.8	N/A
Donor 15	Pre‐Treat	Female	Caucasian	60	98.3	M0
Donor 16	Pre‐Treat	Male	Caucasian	38	85.2	M5a
Donor 17	Pre‐Treat	Male	Caucasian	43	96	M5a
Donor 18	Pre‐Treat	Female	Caucasian	28	95.1	M4
Donor 19	Pre‐Treat	Female	Caucasian	63	80	M3
Donor 20	Pre‐Treat	Female	Caucasian	86	86	M5a
Donor 21	Pre‐Treat	Female	Caucasian	57	80	N/A
Donor 22	Pre‐Treat	Female	Caucasian	64	80	M5
Donor 23	Pre‐Treat	Female	Caucasian	77	100	M1
Donor 24	Pre‐Treat	Male	Caucasian	63	95	N/A
Donor 25	Pre‐Treat	Female	Caucasian	72	85	M4
Donor 26	Pre‐Treat	Male	Caucasian	71	56	M4
Donor 27	Pre‐Treat	Male	Caucasian	61	76	M2
Donor 28	Pre‐Treat	Female	Caucasian	77	90	N/A
Donor 29	Pre‐Treat	Female	Caucasian	49	75	N/A
Donor 30	Pre‐Treat	Female	Caucasian	70	76	M2
Donor 31	Pre‐Treat	Female	Caucasian	70	72	M6
Donor 32	Pre‐Treat	Female	Caucasian	75	81	N/A
Donor 33	Pre‐Treat	Female	Caucasian	79	67.6	M2
Donor 34	Pre‐Treat	Female	Caucasian	87	92	N/A
Donor 35	Pre‐Treat	Female	Caucasian	81	75	N/A
Donor 36	Pre‐Treat	Female	Caucasian	78	91.4	M0
Donor 37	Pre‐Treat	Male	Caucasian	38	90	M0‐1
Donor 38	Pre‐Treat	Female	Caucasian	68	71	N/A
Donor 39	Pre‐Treat	Male	Caucasian	68	81.1	M1
Donor 40	Pre‐Treat	Female	Caucasian	67	82.5	M4
Donor 41	Pre‐Treat	Male	Caucasian	70	74.6	M1
Donor 42	Pre‐Treat	Male	Caucasian	65	84.4	M2
Donor 43	Pre‐Treat	Male	Caucasian	50	89.2	M5a
Donor 44	Pre‐Treat	Female	Caucasian	46	80	M0
Donor 45	Pre‐Treat	Female	Caucasian	72	92	N/A
Donor 46	Pre‐Treat	Male	Caucasian	75	84	N/A
Donor 47	Pre‐Treat	Female	Caucasian	60	90	M5a
Donor 48	Pre‐Treat	Male	Caucasian	56	78.4	M5a
Donor 49	Pre‐Treat	Female	Caucasian	57	65	M5a

### 2.4 Immunostaining of cells in matrigel

Cells were washed 3 times with PBS on the automated Hamilton liquid handler (Hamilton Company) and subsequently fixed with 4% paraformaldehyde followed by permeabilization in 4% formaldehyde containing 0.5% Triton X‐100. After 3 washes with PBS, cells were blocked in Odyssey blocking buffer. Then, cells were stained with either FITC‐conjugated‐ anti‐human MPO Ab or anti‐human TDT, or anti‐human CD71, or anti‐human CD110, in the presence of 5 μg/mL Hoechst and 2.5 μg/mL cell mask deep red dyes at 4°C overnight. IgG isotype or the absence of primary Ab was used as background staining controls. Next day, plates were washed three times with PBS containing 0.05% tween‐20, followed by one wash with PBS, and cells were imaged under fluorescence microscope at 10× magnification.

### 2.5 Tartrate‐resistant acid phosphatase (TRAP) staining

Cells were washed three times with PBS and fixed with 4% paraformaldehyde at room temperature without permeabilization. Following three more washes with PBS, chromogenic substrate was added according to manufacturer's instruction. Cells were incubated for 20‐60 minutes at room temperature and visualized at 10× magnification to determine best colour development timing for imaging.

### 2.6 Alkaline phosphatase (AP) staining

Cells were washed three times with PBS and fixed for 1 minute with 4% formaldehyde. Following another three washes with PBS /0.05% tween‐20, 20 μL of substrate (one BCIP/NBT tablet in 10 mL of water) was added. Optimal colour development was observed under microscope every 2‐3 minutes, followed by three washes with PBS/0.05% tween‐20. Cells were stored in 30 μL PBS for imaging.

### 2.7 Adipocyte staining

Cells were washed three times with PBS, followed by fixation with 4% formaldehyde and permeabilization in 4% formaldehyde/0.4% Triton X‐100. After three washes with PBS, cells were stained with 5 μg/mL Hoechst and 2.5 μg/mL deep red cell mask dyes for 1 hour at room temperature. Cells were washed three more times with PBS, and lipid tox was added to the plate. After shaking at room temperature for 30 minutes, cells were imaged at 10× magnification.

### 2.8 Mineralization staining

Cells were washed three times with PBS, followed by fixation with 4% formaldehyde for 30 minutes. After three washes with distilled water, cells were stained with Alizarin Red Stain solution for 45 minutes in dark with gentle shaking. After additional four washes with distilled water, cells were stored in PBS and imaged at 10× magnification.

### 2.9 DNA/RNA extraction from AML samples

For dual DNA/RNA extraction from frozen AML samples, cells were lysed with ice‐cold Ambion lysis/binding buffer and the lysates went through a QIA shredder spin column twice. The lysates were then transferred to an AllPrep DNA spin column. Following centrifugation at room temperature, DNA was retained in the spin column and RNA released in the flow through. DNA was extracted according to AllPrep Qiagen instruction. Total RNA was extracted using mirVana miRNA isolation kit following manufacturer's instructions.

For RNA extraction from the 3D gel cultures, cells were first harvested from matrigel using organoid harvesting solution (Amsbio), followed by RNA purification with Qiagen RNeasy plus micro kit according to manufacturer's instruction. RNA integrity and concentrations were assessed using Agilent RNA 6000 pico assay and analysed on Agilent Bioanalyzer 2100.

### 2.10 RNA sequencing (RNA Seq) and data analysis

RNA samples were sent to BGI Hong Kong Co. Limited for RNAseq (100 bp, PE, 8 Gb raw data) using Illumina HiSeq3000/4000 platform. The Agilent TruSeq stranded total RNA kit was used for library preparation according to the manufacturer's instructions (Illumina). Alignment and differential gene expression analysis was performed in Omicsoft Array Studio version 10.0.1.96. Reads were aligned to the human B38 genome reference by using the Omicsoft Aligner, with a maximum of two allowed mismatches. Gene level counts were determined by the OSA algorithm as implemented in Omicsoft Array Studio and using Ensembl.R86 gene models. Approximately 85% of reads across all samples mapped to the human genome (corresponding to 60‐130 million reads). Differential gene expression analysis was performed by the DESeq2 algorithm as implemented in Omicsoft Array Studio with the samples from the cryopreserved, or vehicle‐treated groups serving as reference. A cut‐off of 20 normalized counts in any replicate group was applied when identifying a gene signature to remove genes with very low expression. FusionCatcher (V1.00)^14^ and EricScript (0.5.5),^15^ using default settings, were used to identify candidate fusion transcripts from the RNAseq data. The fusion transcripts identified from FusionCatcher were removed if their fusion descriptions indicate fusion genes of high or very high probability of being a false‐positive fusion transcript. Predicted gene fusions from EricScript with EricScore >0.5 were retained. Gene fusions identified by both the two tools were taken as the final gene fusions in our analysis. GO term enrichment analysis was performed using the PANTHER overrepresentation test (http://pantherdb.org).

### 2.11 WES and data analysis

DNA samples were sent to BGI Hong Kong Co. Limited for WES (100 bp, PE, 7 Gb raw data) using Illumina HiSeq4000 platform. The Agilent SureSelect human All Exon V5 Kit was used for target region capture. For WES variant detection, sequence reads were aligned to human reference genome GRCh38 by bwa mem.^16^ Picard (v1.114) and GATK (Genome Analysis Toolkit, v4)^17^ were applied to post‐process the BAM file including marking duplicates and recalibrating base quality scores to generate analysis‐ready BAM files for variant calling. GATK4 MuTect2^18^ tumour only mode was applied to call the somatic mutations for tumour samples due to the absence of matched normal samples for these AML donors. Whole‐exome sequencing data from 50 normal blood samples were randomly selected as a pool of normal (PoN) controls. Variants called by MuTect2 that present in the Single Nucleotide Polymorphism Database (dbSNP, v151)^19^ while not in the Catalogue of Somatic Mutations in Cancer (COSMIC, v81)^20^ were removed. Variants that have mutant allele depth <4 and total reads depth <15 were excluded to ensure the reliability of data. Variants were annotated with their most deleterious effects on Ensembl transcripts with Ensemble VEP (Variant Effect Predictor, Version 92)^21^ on GRCh38.

## 3 RESULTS

### 3.1 Establishment of a 3D BMMC culture system

**Figure 1:**
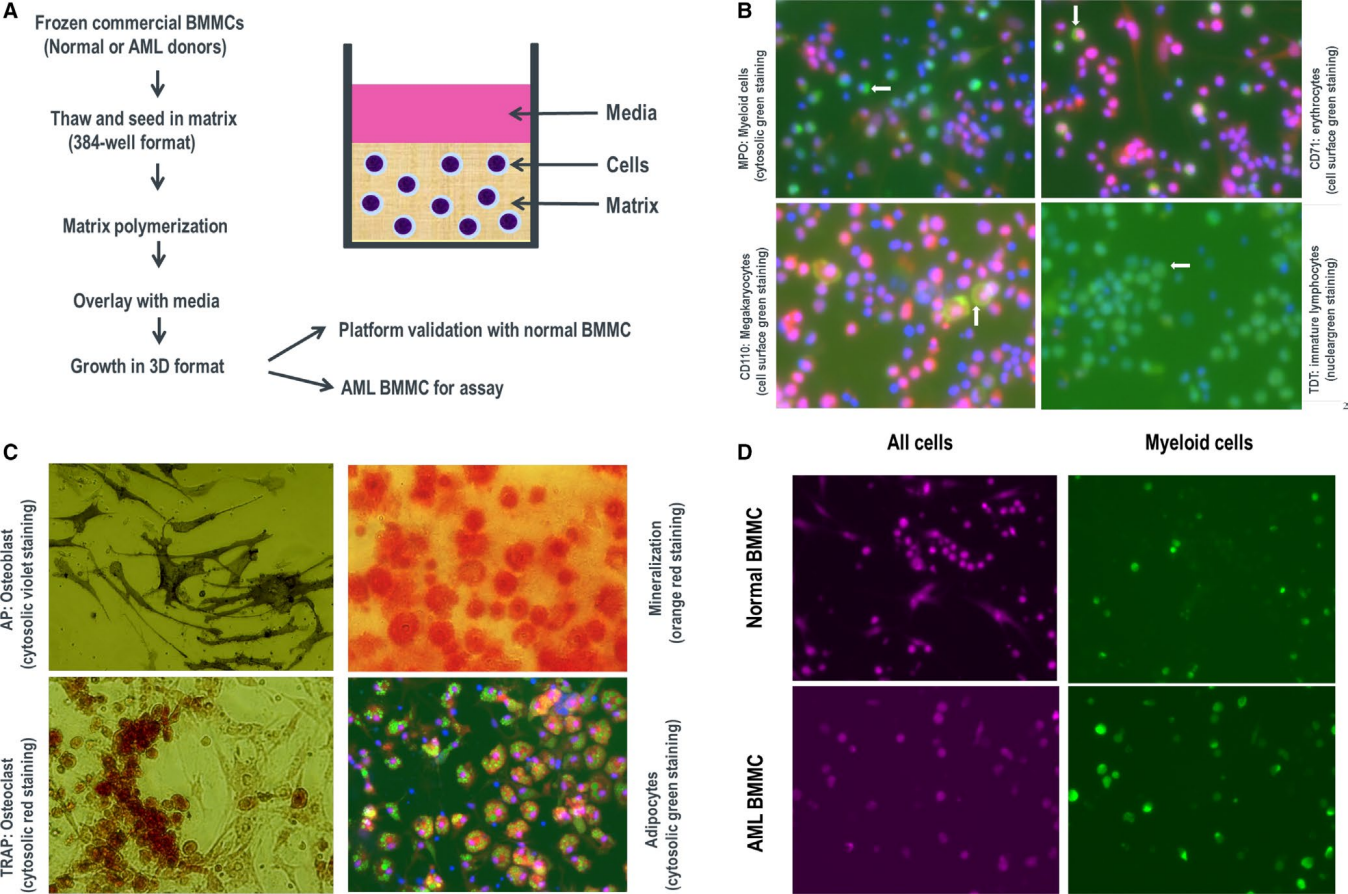
Establishment and characterization of a 3D ex vivo platform for culturing BMMCs from healthy donors and AML patients. A, Workflow of setting up BMMC 3D platform. B, Differentiation and identification of myeloid cells, erythrocytes, megakaryocytes and immature lymphocytes. Blue (Hoechst), staining for nucleus; red (CellMask), staining for cytoplasm; green, staining for target protein. C, Differentiation and identification of osteoblast, mineralization, osteoclast and adipocytes. D, Comparison of the percentage of MPO positive population between a healthy donor and an AML patient

3D culture system was based on the previously reported culture conditions as described by Parikh et al^9^ with additional critical modifications, including replacing patients' autologous serum with 20% FBS and adding a cocktail of 10 cytokines important for maintaining the growth and differentiation of hematopoietic stem cells.^22^, ^23^, ^24^, ^25^, ^26^, ^27^ To enable high throughput drug sensitivity screening, assays were set up in 384‐well plate format as outlined in Figure 1A. To evaluate whether the platform is appropriate for growth and differentiation of BMMCs, samples from three normal donors were tested. Markers characteristic for several lineages of blood and stromal cells were selected for 3D in‐gel immunofluorescence imaging. AF488 fluorophore‐conjugated antibodies were used to identify cell–type‐specific marker proteins. Myeloperoxidase (MPO) was used to identify the subset of granulocytic and monocytic cells (Figure 1B, top left); CD71 for the presence of erythrocytes (Figure 1B, top right); CD110 for megakaryocytes (Figure 1B, bottom left); and terminal deoxynucleotidyl transferase (TDT) for immature lymphocytes (Figure 1B, bottom right). Stromal cells could also be differentiated: osteoblasts were identified by AP staining (Figure 1C, top left). Furthermore, osteoblasts were capable of secreting calcium phosphate and going through mineralization (Figure 1C, top right); Osteoclasts were identified by TRAP staining (Figure 1C, bottom left); Marrow adipocytes were also detected (Figure 1C, bottom right). These results indicate that the defined 3D cultures can mimic bone marrow microenvironment. In contrast to normal donors for which only a subset of cells stain positive for MPO (Figure 1D, top panels), the majority of BMMCs from an AML donor express MPO (Figure 1D, bottom panels). The stromal component varies dramatically among these AML donors. For example, donor 3 has almost no osteoblast present and hardly any mineralization, low percentage of osteoclasts and limited number of adipocytes; donor 16 has a large percentage of osteoblasts and very strong mineralization, low percentage of osteoclasts and high load of adipocytes; donor 47 has many osteoblasts but was incapable of mineralization, with no osteoclast and adipocytes (Figure S1).

### 3.2 Characterization of the growth of AML patient BMMCs in the 3D platform vs 2D culture

**Figure 2:**
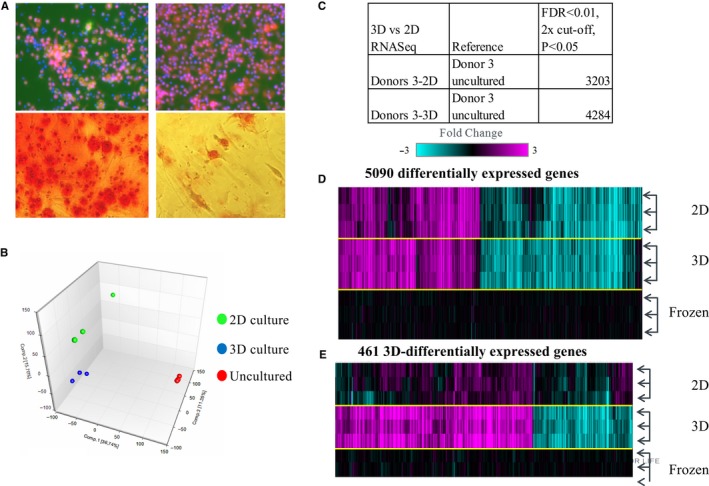
Comparison of 3D platform vs 2D culture. A, Thrombopoiesis and mineralization in 3D vs 2D. B, Summary of gene expression analysis of 3D and 2D vs uncultured samples. Shown in the principal component analysis (PCA) plot are the first three components indicating that the 3D and 2D cultured samples from the same donor cluster closer together than the uncultured samples. C, Differentially expressed transcripts in 3D, 2D vs. uncultured samples meeting the ±2‐fold change and FDR_BH (False Discovery Rate Benjamini & Hochberg) *P* < .01 cut‐off. D, The heat map contains the 5090 genes that form the union signature of 3D and 2D vs uncultured signatures as shown in B. Depicted are the fold change values of each individual sample vs the pool of uncultured samples as baseline. E, The heat map shows 461 genes that are differentially expressed in 3D vs 2D and uncultured samples. The genes were significantly differentially expressed in the 3D vs uncultured comparison and not in the 2D vs uncultured comparison. The genes were also significantly differentially expressed between the 3D vs 2D comparison (using the same cut‐off as in B). See supplemental Excel File for the associated gene lists

Acute myeloid leukaemia bone marrow mononuclear cells that robustly proliferated in 3D platform was also capable of growth in 2D culture with the supplemental cytokine cocktail in culture media. To elucidate physiological advantages of 3D culture, 3D vs 2D cultures were compared for different biological processes using the three healthy donors' BMMCs. Data indicate that the majority of bone marrow functions occur in 2D culture with the exception of thrombopoiesis and mineralization. The presence of megakaryocytes was only detected in 3D culture and was absent in 2D culture for all three normal subjects (Figure 2A top panels and data not shown). Some degree of mineralization could still occur in 2D culture but was greatly reduced (Figure 2A bottom panels and data not shown).

To further investigate the differences between 2D and 3D cultures, gene expression signatures were compared by RNAseq using BMMCs from AML donor 3 after 10‐day culture. 2D and 3D signatures were also compared to uncultured cryopreserved samples from the same donor. Results of this study indicate that, the gene expression patterns of 3D and 2D cultures are more similar to each other than to the uncultured frozen samples (Figure 2B). A total of 4284 and 3203 genes were differentially regulated in 3D vs 2D system, respectively, when using uncultured samples as a reference (Figure 2C). When looking at the union signature (5090 genes), the majority of differentially expressed genes as compared to the cryopreserved sample are similar in both 2D and 3D systems (Figure 2D). However, a cluster of 461 genes was significantly distinct in 3D platform as compared to the 2D system, indicating existence of additional functionalities in the 3D environment (Figure 2E). Several Gene Ontology (GO) terms were over‐represented in this 461 gene set (supplemental gene and GO excel file), including MHC class II complex assembly, heme metabolic processes and cytoskeletal processes. Examples of genes involved in these pathways include ALAD, ALAS2, CPOX, FECH and HMBS (heme biosynthesis), COL18A1 (fibrosis), and HLA‐DMA, HLA‐DMB and HLA‐DPA1 (antigen presentation). The genes in the 4284, 3203, 5090 and 461 gene sets are also listed in the supplemental Excel File.

### 3.3 Determination of the Ara‐C response in 49 AML patients

**Figure 3:**
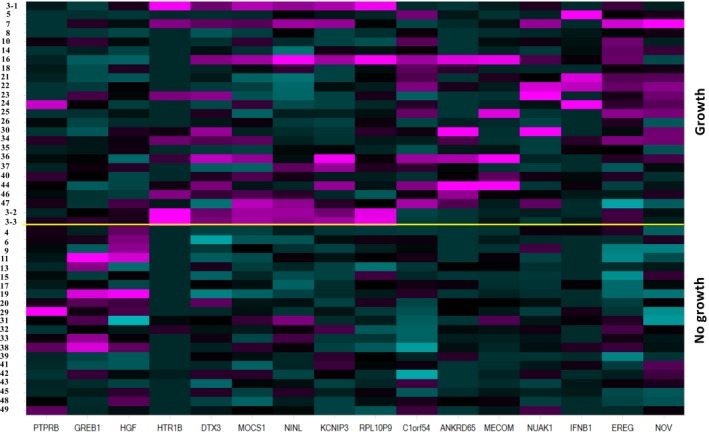
Gene signature that differentiates AML donors with robust growth vs no or poor growth. Shown in the heat map are the 16 genes that are significantly differentially expressed (±2‐fold change and FDR_BH *P* < .01) between the growth and no growth groups. Depicted are the centre scaled FKPM upper quartile normalized (UQnorm) log2 transformed values (colour gradient is −2.5 to 2.5). See Supplemental Excel file for the associated gene list

**Figure 4:**
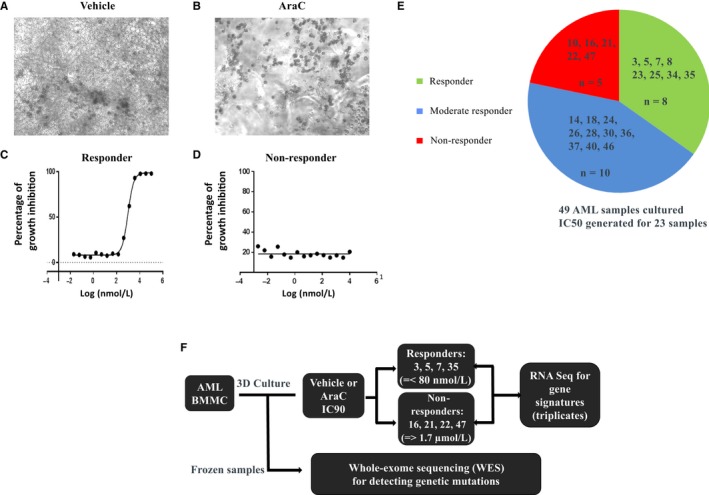
Treatment of AML donors with Ara‐C. A, A responsive donor 8 treated with DMSO; B, The same responder treated with 10 μmol/L Ara‐C; C, A dose‐response curve from donor 8; D, A dose‐response curve from a non‐responder donor 10. Images were taken under 10× magnification. E, The pie chart of distribution of Ara‐C responders, moderate and non‐responders. F, Experimental work flow chart to identify prognostic gene signatures and genetic biomarkers

The newly defined 3D platform was then used to culture BMMCs from 49 AML donors (Table 1) representing various disease states and subtypes. Despite addition of the 10‐cytokine cocktail, only twenty‐four AML bone marrow samples exhibited robust ex vivo growth. To understand whether certain intrinsic gene signatures are associated with AML cell growth, RNA samples from the original cryopreserved BMMCs were analysed by RNAseq. A small cluster of 16 genes were differentially expressed between the no growth and growth group (Figure 3, and the supplemental Excel File). A 15‐point 3‐fold dilution of Ara‐C response curve (top concentration 10 μmol/L) was obtained for 23 donors, and IC50 values were summarized in Table 2. IC50 values ranged from 17 nmol/L to higher than 10 μmol/L. Ara‐C responsiveness in 2D vs 3D culture was also compared using nine donors, and IC50 value shifts of more than 10‐fold were observed for three donors (Table 2) with the relative IC50 ranking being different in 2D vs 3D platform, suggesting that microenvironment affects drug response. Figure 4 represents bright field light microscope images of a representative responder treated with either vehicle (Figure 4A) or 10 μmol/L Ara‐C (Figure 4B) and IC50 curves of the same responder (Figure 4C) and a non‐responder (Figure 4D).

**Table 2:** AML patient IC50 in response to 10‐day Ara‐C treatment

AML donors	IC50 (nmol/L) 3D culture	IC50 (nmol/L) 2D culture
Donor 8	IC50 = 17	
Donor 3	IC50 = 31.59	IC50 = 11.79
Donor 5	IC50 = 43.66	IC50 = 654.6
Donor 25	IC50 = 61	
Donor 35	IP = 55.9, IC50 = 63	
Donor 7	IC50 = 80.41	
Donor 34	IC50 = 84	
Donor 23	IC50 = 95	IC50 = 5.5
Donor 24	IC50 = 143	IC50 = 137
Donor 28	IC50 = 162	
Donor 14	IC50 = 182.9	
Donor 30	IC50 = 233	
Donor 26	IC50 = 239.9	IC50 = 169.9
Donor 36	IC50 = 243	
Donor 46	IC50 = 270	
Donor 37	IC50 = 342.3	IC50 = 208.5
Donor 40	IC50 = 503.5	
Donor 18	IC50 = 708.7	
Donor 22	IC50 = 1706	IC50 = 278
Donor 47	IC50 = 1857	IC50 = 1632
Donor 16	IC50 = 8700	IC50 = 771.8
Donor 21	IP = 127, IC50 > 10 000	
Donor 10	IC50 > 10 000	

### 3.4 Determination of gene signatures that predict AML patient responsiveness to Ara‐C

**Figure 5:**
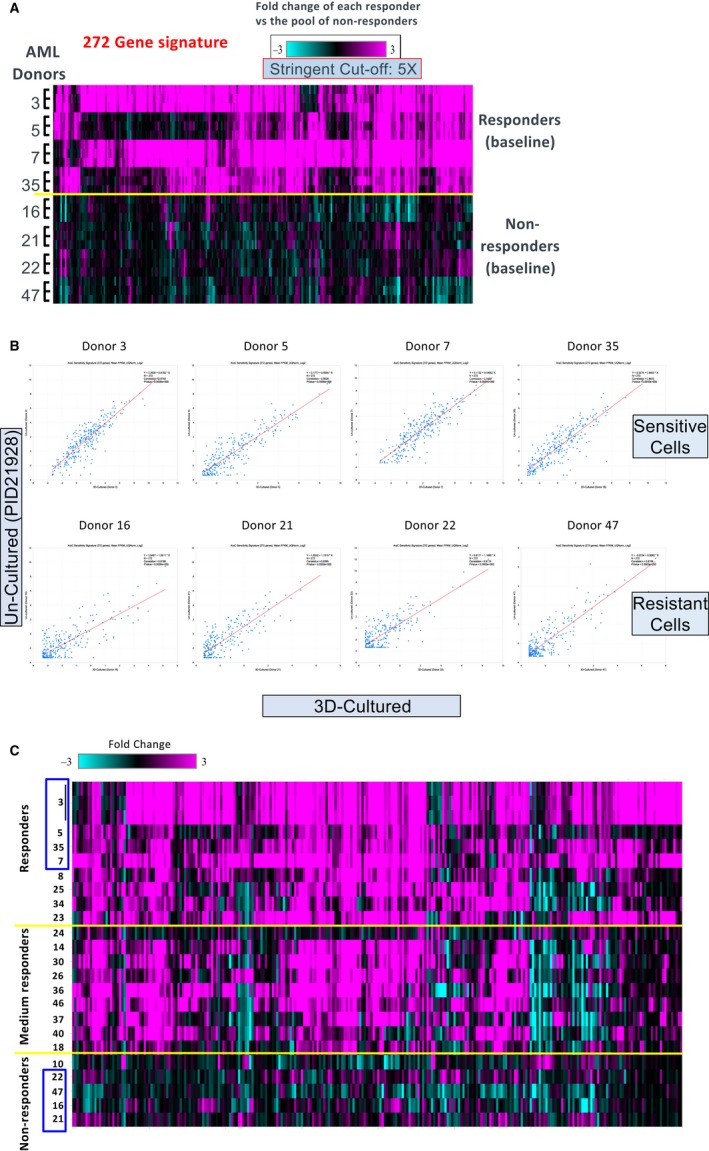
The gene signature in vehicle‐treated Ara‐C responders. A, The heat map shows the 272 genes that are significantly and differentially expressed (at least 5‐fold up‐regulated with FDR_BH <0.01) in four responders vs four non‐responders at baseline 3D culture (DMSO treatment). Depicted are the fold change values of each individual sample vs the pool of non‐responders at baseline. B, Confirmation of the 272 gene signature in the four uncultured cryopreserved responder samples. Depicted in the scatter plots in B are the FPKM_UQnorm_Log2 values for the 272 genes, as shown in A, across the 3D and uncultured frozen samples for the same eight donors. C, Confirmation of the 272 gene signature in eight uncultured responder samples, nine moderate responder samples and 5 non‐responder samples. Shown in the heat map in C are the fold change values for the 272 genes, as shown in A, across all 22 uncultured frozen samples (each individual sample vs the pool of the non‐responders as baseline)

The 23 tested AML donors were classified as 8 responders (IC50 < 100 nmol/L), 5 non‐responders (IC50 ≥ 1.7 μmol/L) and 10 moderate responders (100 nmol/L < IC50 < 1.7 μmol/L) as indicated in Figure 4E. To investigate whether certain gene signatures can be used to predict the responsiveness of AML donors to Ara‐C, a RNAseq experiment was designed as shown in Figure 4F. Donors 3, 5, 7 and 35 were selected as responders and donors 16, 21, 22 and 47 were chosen as non‐responders. The BMMCs of these eight donors were seeded in 3D platform and treated with Ara‐C at their respective IC90 concentrations the following day for 10 days. IC90 values were extrapolated in GraphPad Prisms 7 for donors 3, 5, 7, 35 and 47 (647, 583, 325, 503, and 4943 nmol/L, respectively). IC90 values could not be calculated for non‐responders 16, 21 and 22, therefore the top concentration of 10 µmol/L was used for Ara‐C treatments. RNAseq analysis revealed a striking difference in gene expression pattern between the two groups treated by vehicle. A cluster of 272 genes was significantly up‐regulated by more than fivefold in responder donors compared to non‐responder donors (Figure 5A). To confirm that this gene signature is not an artifact of 11‐day 3D culture due to the presence of supplemental cytokines, expression levels of these 272 genes were compared to those of the same four responders' cryopreserved samples. The correlation between 3D‐cultured and cryopreserved samples is highly significant (*R* > .8 and *P* < .001) for each of the four tested responders (Figure 5B), indicating that this 272 gene signature is most likely specific for drug response. When tested on independent uncultured frozen samples, this 272 gene signature maintained the same differential expression pattern among responders, moderate responders and non‐responders (Figure 5C). With the inclusion of the additional independent donor samples, there were 96 out of the 272 genes that were still at least 5‐fold up‐regulated (with FDR <0.01) in the uncultured samples and 199 of the 272 genes with at least 2‐fold up‐regulated (with nominal *P* < .05).

**Figure 6:**
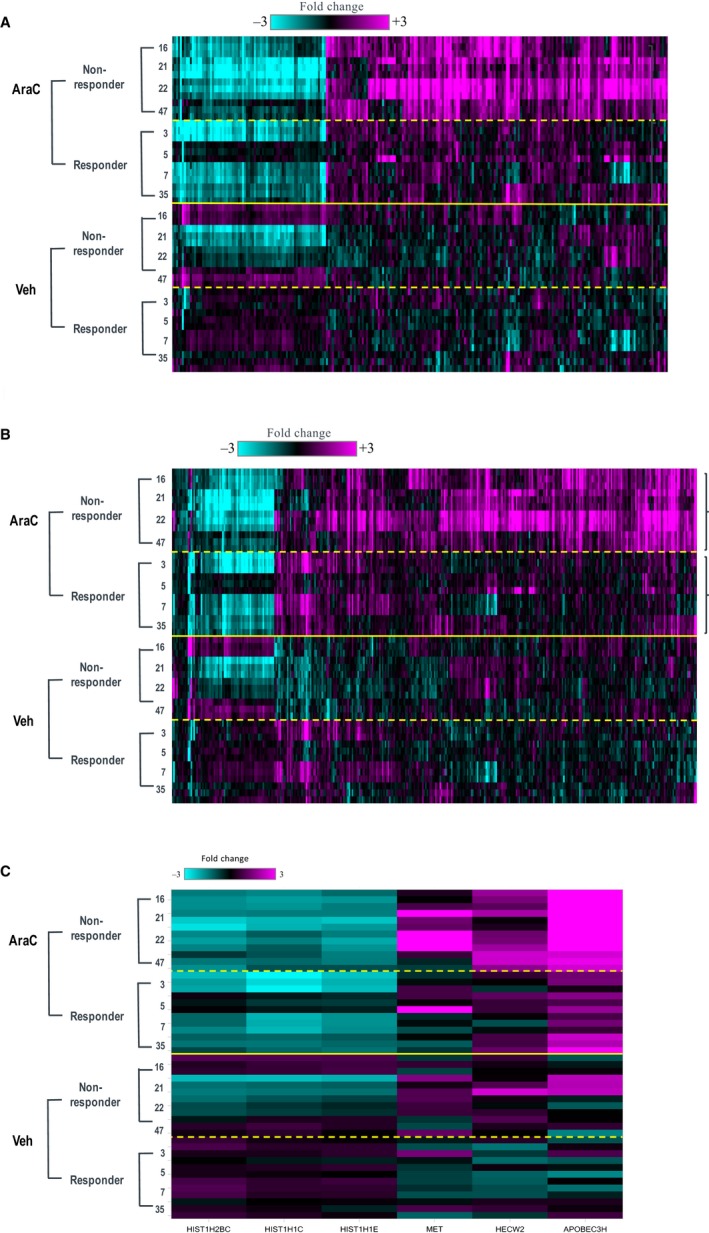
Gene expression analysis following Ara‐C treatment. A, The heat map contains the 248 genes that are significantly differentially expressed (±1.5‐fold change with FDR_BH < 0.01) following Ara‐C treatment compared to vehicle treatment in all 8 donor samples. The four responders and four non‐responders were combined as one group. Depicted are the fold change values of each individual Ara‐C‐treated sample vs the pool of vehicle‐treated samples (eight donors). B, The heat map contains the 365 genes that are significantly differentially expressed (± 1.5‐fold change with FDR_BH < 0.01) following Ara‐C treatment compared to vehicle treatment in the four non‐responders. Depicted are the fold change values of each individual Ara‐C‐treated sample vs the pool of vehicle‐treated samples (eight donors). C, The corresponding heat map of the Ara‐C signature in responder donor samples (six genes)

Upon Ara‐C treatment at IC90 values, a cluster of 248 genes was differentially expressed compared to vehicle‐treated samples, when responder and non‐responder samples were combined (Figure 6A). Interestingly, the transcriptional regulation by Ara‐C treatment was more robust in the non‐responder samples (365 genes, Figure 6B) than in the responder samples (six genes, Figure 6C). Genes involved in several pathways were regulated by Ara‐C either exclusively or more profoundly in non‐responder samples, such as the p53 pathway (for example TP53I3, GADD45A, CDKN1A and MDM2).

### 3.5 Determination of gene fusions and mutations in AML samples

Abnormal gene fusions are frequently observed in AML patients due to chromosomal abnormalities.^3^ In our cohort of samples, a total of 14 gene fusions were identified in 13 AML donors by RNAseq after merging the results from FusionCatcher and EricScript Methods.^14^, ^15^ Table 3 shows the 14 gene fusions and the samples that they occur. Eight out of the 14 gene fusions have been previously reported,^4^, ^28^, ^29^, ^30^ leaving 6 novel ones. To confirm these novel gene fusions, the gene fusion supporting reads were aligned to the fusion sequences to check the break points (supplemental Figure 2).

**Table 3:** Gene fusions identified in used AML samples

Fusion genes	Donor
ABL1‐KIAA1671	Donor 36
AKAP8‐CACNA1A	Donor 31
ALOXE3‐ETV6	Donor 29
*BCR‐ABL1*	Donor 36
*KMT2A‐MLLT10*	Donor 16
*KMT2A‐MLLT3*	Donor 47
LATS2‐HMGB1	Donor 7
*MLLT10‐ATP5L*	Donor 16
*PML‐RARA*	Donor 11
*RUNX1‐DYRK1A*	Donor 19
*RUNX1‐RUNX1T1*	Donor 6, Donor 37
ST6GAL1‐RTP4	Donor 45
TANC2‐ATP2C1	Donor 20
*TPM4‐KLF2*	Donor 34

Note

Gene fusions that have been previously reported are indicated in italics.

For analysis of somatic mutations by WES, the tumour only pipeline was selected because of the presence of more than 80% of malignant blasts in the majority of BMMCs. Fisher exact test was applied to identify mutated genes to differentiate bone marrow sample growth status. Different variants within a gene were merged. If a gene has at least one somatic variant, this gene was assigned as a ‘mutant’; otherwise, it was a ‘non‐mutant’. Only genes mutated in at least two samples were included. Due to the small sample size, these associations are not statistically significant after correcting the *P*‐values with false discovery rate. However, interesting trends were seen from the 12 genes with *P*‐value < .05 (Table 4A). If only the non‐synonymous somatic mutations were involved into the association analysis, only one gene differentiates bone marrow sample growth status with *P*‐value < .05 (Table 4B). Mutations of CEP170 gene were found in 7 out of 22 AML donors with robust growth, whereas no mutation was identified in any of the 21 AML donor samples with poor or no growth. Mutations were enriched in some genes for BMMCs that were not able to grow or grew poorly in the 3D platform such as PHTF1, RAPGEF6, ARHGAP26, POM121L12, DOCK5, SPAG6 and ABCC9, which were found in 4 poor growing samples but were not identified in robust growing samples. Wilcoxon rank‐sum test was applied to examine the Ara‐C responsiveness (log2 transformed of Ara‐C IC50 response value) of mutated genes. The top 20 genes show their trends to differentiate the Ara‐C H50 values and could be used to predict Ara‐C responsiveness (Table 5A). If only the non‐synonymous somatic mutations were counted, five genes (HDAC8, CRYBG3, PRSS3, MYH7 and ZAN) were found to associate with Ara‐C responsiveness with *P*‐value < .05 (Table 5B).

**Table 4:** (A) Genes that differentiate AML BMMC growth and no growth in the 3D platform. (B) Genes that differentiate AML BMMC growth and no growth in the 3D platform (only involve non‐synonymous variants)

Gene	Samples with no or poor growth		Samples with robust growth		Pval Fisher Exact	Adjusted Pval
	Mutation	normal	Mutation	normal		
(A)						
CEP170	0	21	7	15	0.005292	0.696431
AC116618.1	9	12	17	5	0.022368	0.696431
COPG2	6	15	1	21	0.040656	0.696431
RPS3AP34	15	6	9	13	0.043255	0.696431
SLC9B1P1	7	14	14	8	0.045742	0.696431
PHTF1	4	17	0	22	0.048497	0.696431
RAPGEF6	4	17	0	22	0.048497	0.696431
ARHGAP26	4	17	0	22	0.048497	0.696431
POM121L12	4	17	0	22	0.048497	0.696431
DOCK5	4	17	0	22	0.048497	0.696431
SPAG6	4	17	0	22	0.048497	0.696431
ABCC9	4	17	0	22	0.048497	0.696431
(B)						
POM121L12	4	17	0	22	0.048497	0.67263

**Table 5:** (A) Top 20 genes that predict Ara‐C responsiveness. (B) Genes that predict Ara‐C responsiveness (non‐synonymous variants, *P* value < .05)

Gene	Sample normal	AracScore normal	Sample mutation	AracScore mutation	Pval	Pval. adjustment
(A)						
MAN1B1	16	8.971318	6	6.123962	0.005478	0.458069
AK2	18	8.791587	4	5.509074	0.006004	0.458069
SLC4A8	18	7.49691	4	11.33512	0.0096	0.458069
CCDC146	13	7.055575	9	9.840265	0.009696	0.458069
CCDC14	20	7.685472	2	13.28771	0.012951	0.458069
SLC9A5	20	7.685472	2	13.28771	0.012951	0.458069
KAT2A	20	7.685472	2	13.28771	0.012951	0.458069
MAN2B1	20	7.685472	2	13.28771	0.012951	0.458069
PRAMEF20	15	7.394283	7	9.910088	0.013173	0.458069
DEPDC5	19	8.628282	3	5.449168	0.013864	0.458069
ZAN	18	8.736552	4	5.756729	0.018482	0.458069
BX284668.3	20	7.695517	2	13.18726	0.019876	0.458069
SCN10A	20	7.695517	2	13.18726	0.019876	0.458069
TEC	20	7.695517	2	13.18726	0.019876	0.458069
SNX13	20	7.695517	2	13.18726	0.019876	0.458069
ADAM22	20	7.695517	2	13.18726	0.019876	0.458069
NRP1	20	7.695517	2	13.18726	0.019876	0.458069
PARD3	20	7.695517	2	13.18726	0.019876	0.458069
APP	20	7.695517	2	13.18726	0.019876	0.458069
SEC14L3	20	7.695517	2	13.18726	0.019876	0.458069
(B)						
HDAC8	20	8.468636	2	5.456067	0.029717	0.4936
CRYBG3	20	7.813037	2	12.01206	0.033786	0.4936
PRSS3	20	7.876406	2	11.37837	0.043301	0.4936
MYH7	20	7.876406	2	11.37837	0.043301	0.4936
ZAN	20	8.490254	2	5.23989	0.048812	0.4936

Note

AracScore normal: Average IC50 response values (log2 transformed) of the samples without variant in this gene. AracScore mutation: Average IC50 response values (log2 transformed) of the samples with variant in this gene. Pval.adjustment: *P* value after false discovery rate adjustment.

## 4 DISCUSSION

To our best knowledge, this study is the first one to use a 3D platform to culture a cohort of AML bone marrow samples ex vivo and profile gene signatures and genetic mutations that correlate with growth and Ara‐C responsiveness. 3D culture is currently used as a translational platform for drug discovery because of its physiological relevance and better prediction of drug efficacy.^7^, ^8^ Extensive characterization of differentiation of major lineages of blood and stromal cells using bone marrow samples derived from healthy donors demonstrates that the ex vivo 3D platform appropriately mimics the bone marrow microenvironment. We demonstrated that the 3D platform is superior to 2D culture as evident by lack of or attenuated biological processes in the 2D culture that normally occur in bone marrow microenvironment, such as thrombopoiesis and mineralization. Furthermore, RNAseq experiments identified a set of 461 genes that differentially expressed in the 3D platform compared to 2D culture. The 3D culture enables ex vivo drug testing duration within the same time frame of in vivo chemotherapy cycle, making the results more prognostic for predicting in vivo efficacy. This 3D platform has been miniaturized to a high throughput 384‐well plate format, and drug treatments were handled through automated liquid handlers. It is feasible to test multiple drugs for bone marrow samples from naïve AML patients to determine the optimal therapeutic regimen prior to administration of treatments. The accurate calculation of IC50 value of each patient's sample may also assist in determining the in vivo dosage.

Despite our diligent efforts to define the ex vivo growth condition, approximately half of the cryopreserved samples did not proliferate, raising the question whether certain intrinsic features of these specimens prevented ex vivo growth. The hypothesis is supported by WES analysis results which reveal enrichment of certain mutations in non‐proliferating samples and a different mutation in proliferating samples. The 3D platform was further utilized to test the responsiveness to standard AML therapy drug Ara‐C and both responders and non‐responders have been identified. We identified a 272 gene signature that is associated with all eight Ara‐C responders at baseline. The presence of this gene signature has been confirmed in the original cryopreserved samples of the same donors as well as additional independent samples from responders and moderate responders, further validating the reliability of the 3D culture system. We also found that Ara‐C non‐responders have differentially regulated pathways compared to responders. In addition to prognostic gene expression signatures, new gene fusions have been identified with this cohort of patients. Whole‐exome sequencing analysis reveals genetic biomarkers that are associated with ex vivo growth and Ara‐C responsiveness. Gene profiling and genetic markers for predicting AML drug responsiveness have been explored by multiple groups but the gene signatures usually do not overlap,^31^, ^32^, ^33^, ^34^ indicating the complex pathogenesis of the AML disease, when different patient cohorts' analyses result in different outcome. APP ranks 19 in our top 100 gene list for predicting Ara‐C responsiveness and was reported to be one of the potential candidate pathway genes of relevance for pharmacogenetic studies on ara‐C and other nucleoside analogs.^33^ A most recent gene profiling work performed with uncultured and untreated samples presented a large functional genomic data set of primary AML bone marrow mononuclear cells and revealed new markers and mechanisms of drug sensitivity and resistance.^6^ Additionally, the ex vivo drug testing of 122 small molecule inhibitors was done in 2D culture for a short duration of 4 days and did not include Ara‐C. There is about 20% overlap in mutated genes identified from this study compared to ours, indicating genetic markers predicting common drug responsiveness.

## CONFLICT OF INTEREST

All authors are current employees of Merck Sharp & Dohme Corp., a subsidiary of Merck & Co., Inc., Kenilworth, NJ, USA and may own stock or stock options in Merck & Co., Inc., Kenilworth, NJ, USA.

## AUTHOR CONTRIBUTIONS

All authors reviewed the manuscript and approved its content. HX and HC designed the study. HC acquired patient samples and performed sample preparation for profiling. HX performed experiments and drafted the manuscript. ESM, SJ, LC, RC performed data analysis for RNASeq and whole‐exome sequencing. JC and KK were involved in sample handling. MSM, NF, RA, MM, BN, GA and IK had intellectual contributions. GA proposed the idea of setting up the 3D AML platform. IK frequently participated in discussions and made intellectual contributions to experimental design and result analysis.

## Data Availability

This manuscript does not contain sharable data.
