# Supplementary figures and images for: Identification of predictive genetic signatures of Cytarabine responsiveness using a 3D acute myeloid leukaemia model
Source: J Cell Mol Med. 2019 Aug 26;23(10):7063–77. doi: 10.1111/jcmm.14608 (PMC6787505; doi:10.1111/jcmm.14608)

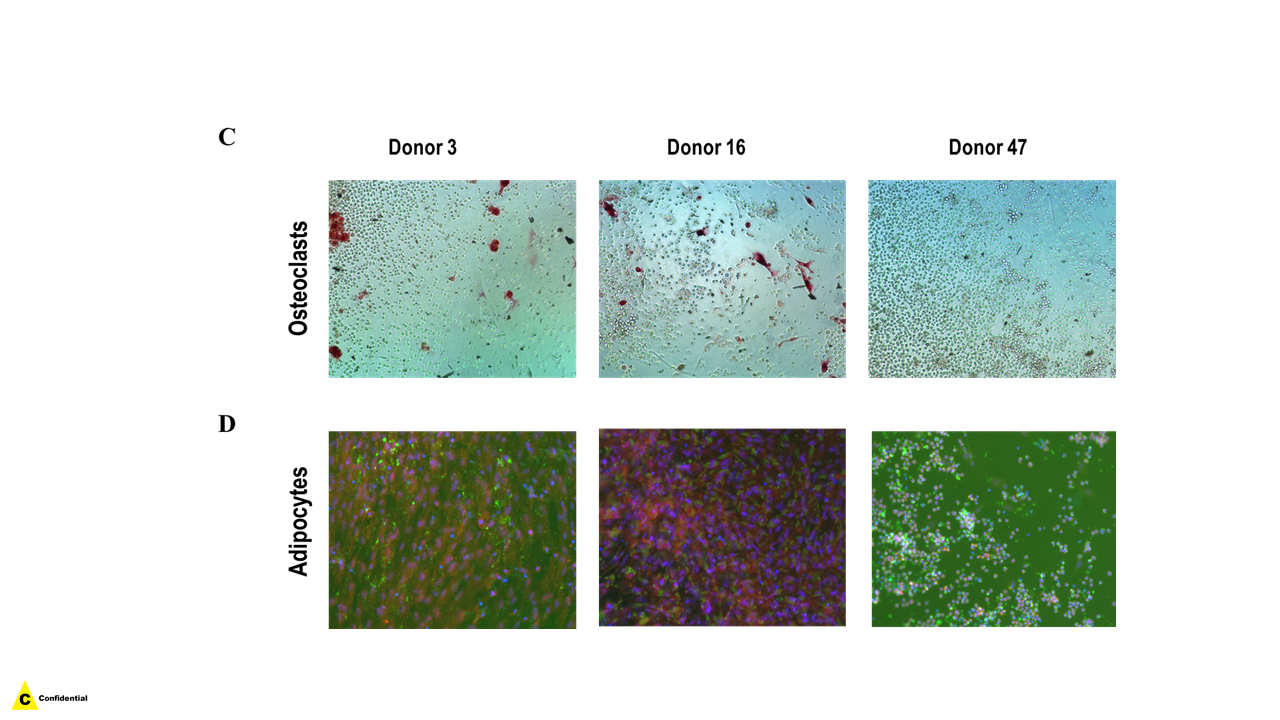

Supplement: Supplementary file 1 [file JCMM-23-7063-s001.zip › Supplemental figure 1C-D.tif]

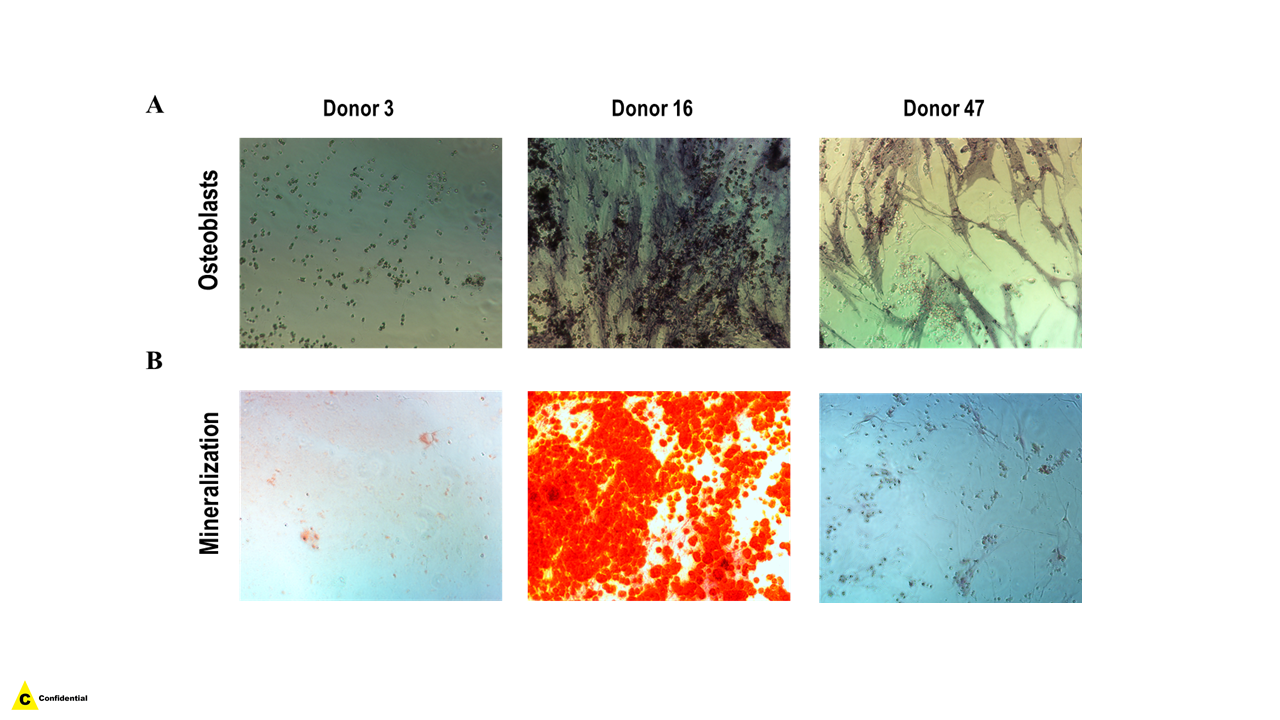

Supplement: Supplementary file 1 [file JCMM-23-7063-s001.zip › Supplemental figure 1A-B.tif]
