# Supplementary material for: Identification of predictive genetic signatures of Cytarabine responsiveness using a 3D acute myeloid leukaemia model
Source: J Cell Mol Med. 2019 Aug 26;23(10):7063–77. doi: 10.1111/jcmm.14608 (PMC6787505; doi:10.1111/jcmm.14608)
Supplement: Supplementary file 2 [file JCMM-23-7063-s002.zip › jcmm14608-sup-0002.docx]

The predicted fusion transcripts and reads supporting the gene fusion were extracted from the results of FusionCathcer for all the novel gene fusions. Reads were aligned to the predicted fusion transcript with Bowtie 2[1]. The sequence alignment was visualized with IGV [2] (Please see below). Since there’s no predicted fusion transcripts of LATS2-HMGB1 and ST6GAL1-RTP4, only the fusion junction sequences were extracted, and reads were aligned to.

ABL1-KIAA1671


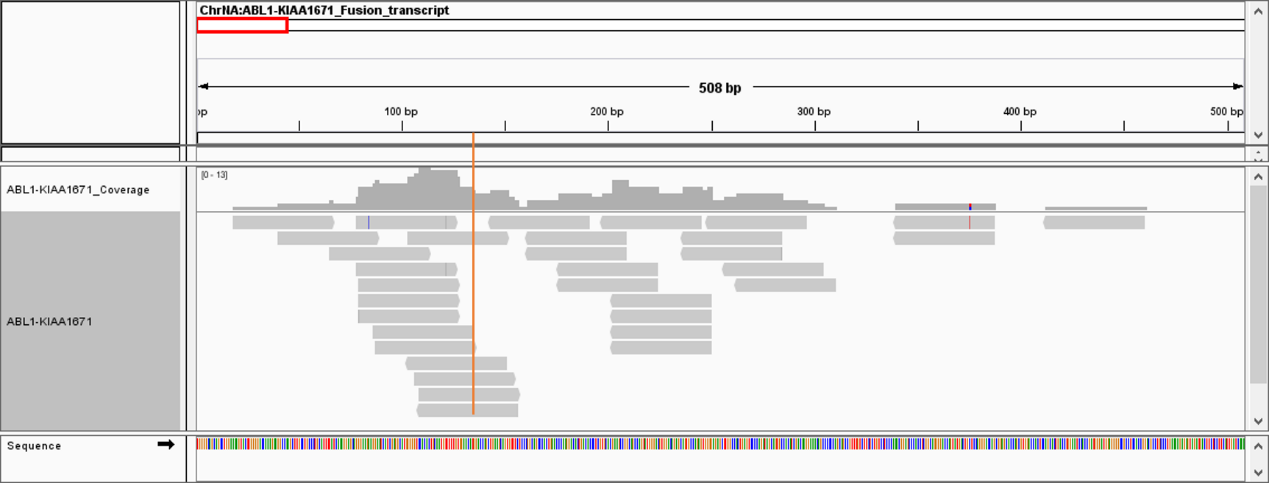


AKAP8-CACNA1A


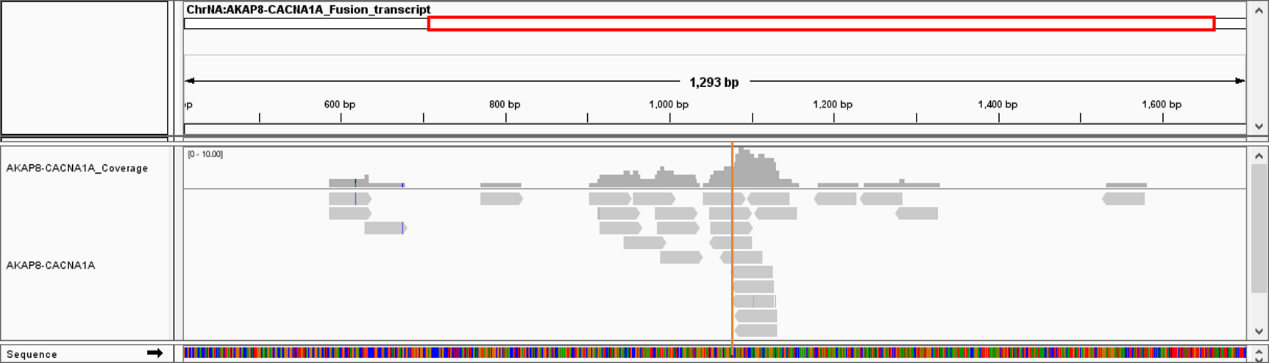


ALOXE3-ETV6


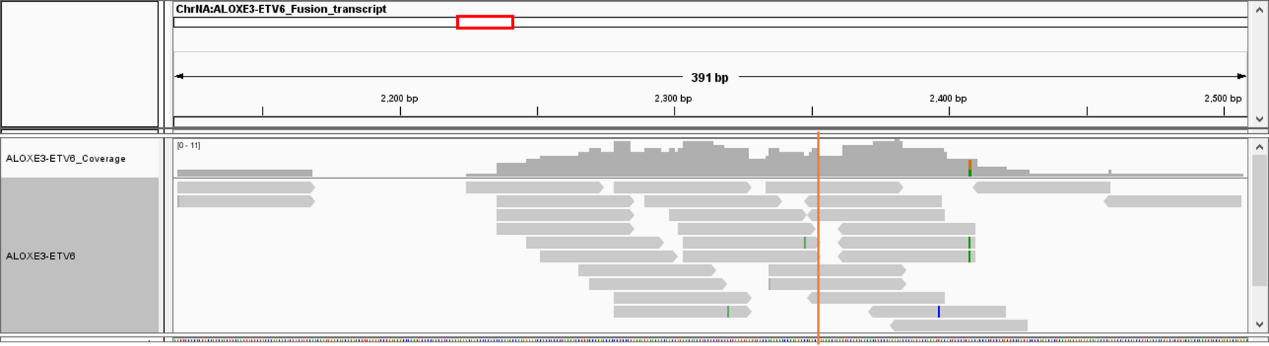


TANC2-ATP2C1


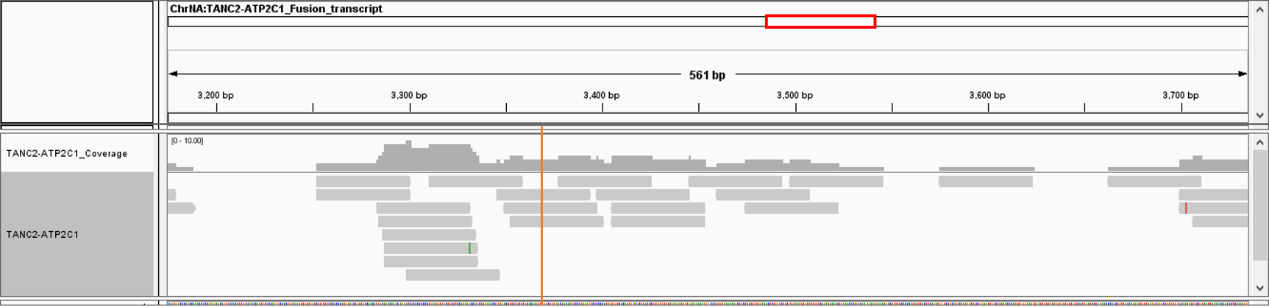


LATS2-HMGB1


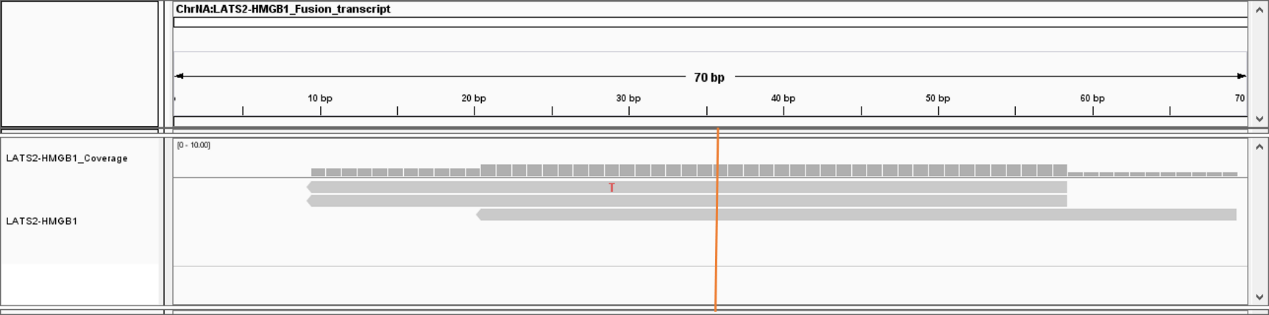


ST6GAL1-RTP4


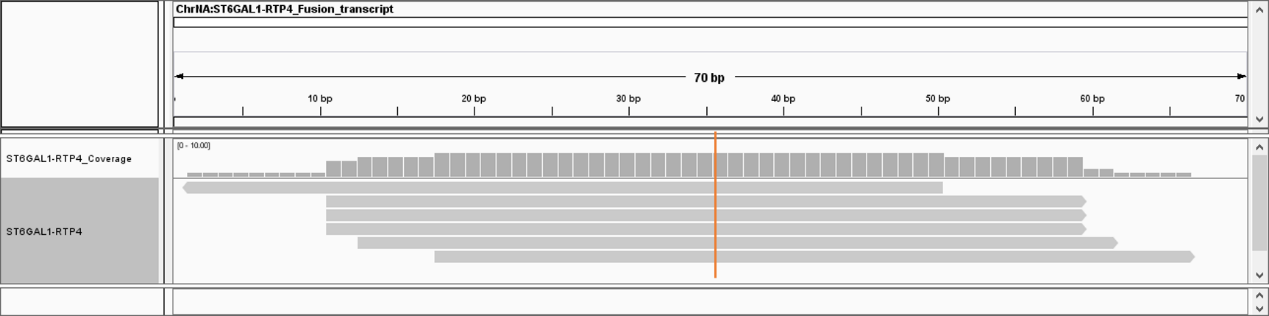


[1] Langmead B, Salzberg S. [Fast gapped-read alignment with Bowtie 2](http://www.nature.com/nmeth/journal/v9/n4/full/nmeth.1923.html). [*Nature Methods*](http://www.nature.com/nmeth). 2012, 9:357-359.

[2] Robinson, James T., et al. "Integrative genomics viewer." *Nature biotechnology* 29.1 (2011): 24.
